# Synthesis and Characterization of Hierarchical ZSM-5 Zeolites with Outstanding Mesoporosity and Excellent Catalytic Properties

**DOI:** 10.1186/s11671-018-2779-8

**Published:** 2018-11-15

**Authors:** Guoqiang Song, Wenting Chen, Peipei Dang, Shengyuan Yang, Yuan Zhang, Yuanyi Wang, Ruidi Xiao, Rong Ma, Fuxiang Li

**Affiliations:** 1 2011 Special Functional Materials Collaborative Innovation Center of Guizhou Province, Guizhou Institute of Technology, 1st Caiguan Road, Yunyan District, Guiyang, 550003 Guizhou Province China; 2 Engineering Technology Research Center of Fluorine Silicon Material, School of Chemical Engineering, Guizhou Institute of Technology, Guiyang, China; 3 Key Laboratory of Light Metal Materials Processing Technology of Guizhou Province, Guizhou Institute of Technology, Guiyang, China; 4 College of Chemistry and Chemical Engineering, Taiyuan University of Technology, No. 79 Yingze West Street, Taiyuan, 030024 Shanxi Province China

**Keywords:** Hierarchical, ZSM-5 zeolites, Soft-template, Benzylation, LDPE cracking

## Abstract

A novel soft-template (ST) is fabricated and successfully employed as mesoporogen to synthesis hierarchical ZSM-5 zeolites with outstanding mesoporosity and high hierarchy factors. The as-produced soft-template can connect steadily with the MFI frameworks by covalent bonds of –Si–O–Si– during the high-temperature hydrothermal crystallization process. This type of connection mode can effectively avoid the formation of amorphous materials, and the specific structure of this soft-template can efficiently introduce plentiful of mesopores with few micropores being consumed. The particles of as-synthesized hierarchical ZSM-5 zeolites are in size of about 1 μm, which are made up of nanocrystals of 60–150 nm. The structure parameters of these samples are characterized with the techniques of X-ray diffraction (XRD), Fourier transform infrared (FTIR) spectroscopy, nitrogen sorption, scanning electron microscope (SEM), transmission electron microscope (TEM), NH_3_ temperature-programmed desorption (NH_3_-TPD) and thermogravimetric (TG). Due to the nature of zeolites and great microporosity, these hierarchical samples present great tolerance of hydrothermal treatment. And because of the intracrystalline mesopores, large external surface areas, and abundant accessible acid sites, whether in conversion rate of reactants or selectivity of products, the hierarchical samples exhibit excellent catalytic performance in the reactions of alkylation between benzene and benzyl alcohol, cracking of 1,3,5-tri-isopropylbenzene, and thermal cracking of low-density polyethylene (LDPE), respectively.

**Electronic supplementary material:**

The online version of this article (10.1186/s11671-018-2779-8) contains supplementary material, which is available to authorized users.

## Background

Zeolites are widely used as adsorbents, ion exchangers, and heterogeneous catalysts in a variety of applications, due to the abundant surface acidity, large surface area, excellent hydrothermal stability, and particular molecule sieving ability [1]. However, the diffusion limitations of bulky reactant molecules in crystals are a very severe problem during catalysis reactions, because of the small and sole even partial occlusive micropores in conventional zeolites [2].

Although ordered mesoporous materials are synthesized [3] to solve the problem of diffusion limitations [4], these materials are of amorphous frameworks in essence, having poor surface acidity and unstable structure property, which bring about unsatisfied activity in acid catalyzed reactions [5]; thus, it is obviously difficult to improve the catalysis performance simply through the production of analogous materials to settle the diffusion issues [6]. In consideration of the importance of zeolite frameworks, the nanosized zeolites with short intracrystalline path length have been produced to solve the diffusion problem, whereas it is hard to recycle these nanocatalysts after heterogeneous catalysis [7] and the hydrothermal stability of nanosized zeolites is also worthy of discussion [8]. Therefore, introducing a secondary pore system besides the micropores in zeolite crystals is becoming a major research hotspots [9, 10] and that is the synthesis of hierarchical zeolites.

These hierarchical zeolites possess the advantages of traditional zeolites and mesoporous materials, which can greatly expand their applications in catalysis, benefiting from the increased external surface area, abundant surface acid sites, decreased diffusion path length, and great hydrothermal stability as well [11–13]. It was proved that the deposition of coke preferred occur in mesopores rather than in micropores in catalysis applications [14].

The chemical etching in traditional zeolite crystals is an attractive method to obtain hierarchical zeolites, including dealumination and desilication; however, the mesopores etched by dealumination are commonly of intercrystalline, and the desilication always leads to the decrease of the crystallinity and the hydrothermal stability as well [15], and the most important is that the nature of chemical etching is to strip the atoms of frameworks and will destroy the stability of structure and surface acidity seriously [10]. The templating approaches can induce the formation of mesopores avoiding the damage of framework properties to a large extent [16, 17]. Lots of hard templates (activated carbon, carbon fibers, aerogels, and polymer aerogel) and soft templates (cationic polymers, amphiphilic organosilane surfactants, and silylated polymers) have been proved its applications in production of hierarchical zeolites [8, 11, 13, 18]. Actually, due to the characteristic of hydrophobicity, the mesopores induced by the carbon hard templates always have too large mesopore size and too broad pore size distribution, which go against with the products selectivity during catalysis reactions [19]. Moreover, the soft templates by virtue of chemical atomic connections or charge compensation with the frameworks of zeolites, it comes true that the pore parameters of the produced hierarchical zeolites become adjustable and multiple. F. S. Xiao et al. have produced hierarchical zeolites with excellent catalytic performance by employing cationic polymers as mesoporogen [20]. M. Choi et al. have invented a way of preparing hierarchical zeolites with uniform mesopores by utilizing a rationally designed amphiphilic organosilane surfactant [21–23]. Hui Wang et al. synthesized hierarchical ZSM-5 having small intracrystal mesopores by employing a silane-functionalized polymer [24]. Nevertheless, these complicated mesoporogens have the risk to be dislodged from the zeolite frameworks during the crystallization process and finally obtain the hybrid materials of conventional zeolites and amorphous substances, because of the unsteadily connection mode between templates and frameworks [25]. Moreover, it is difficult to fabricate the soft-mesoporogens mentioned above accurately because of their intricate structure, which greatly restrict the industrial production.

Recently, lots of works have been reported in synthesizing of hierarchical zeolites of different crystalline-type by various methods and their applications in many fields with great performance and potentiality. Sergio Fernandez et al. have synthesized hierarchical beta zeolites with rational selection of pore-directing agents, and a top-down base leaching method has been demonstrated by the feasibility of tailoring the mesopore structures [26]. Hengbao Chen et al. produced hierarchical ZSM-5 zeolites with cetyltrimethylammonium bromide (CTAB) containing silicalite-1 as seed, and the methanol to propylene (MTP) reaction was employed to investigate the catalytic performance of the as-obtained zeolite samples, which exhibited a comparable activity with the samples reported in literature [27]. Saros Salakhum et al. have greenly synthesized hierarchical faujasite nanosheets from renewable resources of corn cob ash-derived nanosilica and as efficient catalysts for hydrogenation of lignin-derived alkylphenols, and the high yield of 4-propylcyclohexanol over this novel designed catalysts was about 2.14 times compared with the conventional faujasite [28]. Xiao-Lin Luo et al. have synthesized hierarchical porous materials with various structures by controllable desilication and recrystallization in the presence of microwave. Partial removal of the template from the micropores provided an opening framework in ZSM-5 for subsequent desilication in alkaline solution by microwave digestion. The surface area and pore volume of the hierarchical porous materials were enhanced greatly compared with those of the pristine ZSM-5 because of the large contribution of the mesopores [29]. Yanming Jia et al. have also synthesized hierarchical ZSM-5 zeolite via dry gel conversion-steam assisted crystallization process, and the catalysts exhibited significantly high catalytic lifetime and selectivity of light aromatics (benzene, toluene, and xylene) in aromatization of methanol [30]. Li Peng et al. have fabricated novel hierarchical ZSM-5 zeolite membranes with tunable mesopores by using amphiphilic organosilane 3-[(trimethoxysilyl) propyl]octyldimethyl-ammonium chloride as the mesogenous template, and these membranes have a great potential for ultrafiltration with high performance [31].

In conclusions, it is critical that the mesoporogen not only should have hydrophobic groups to expand space to provide conditions for the formation of mesopores, but also should have a steadily connection method with zeolite precursor during the process of high-temperature crystallization [21]. The as-obtained soft-template in this work is designed to possess a ternary ammonium in the center which is connected with three hydrophobic short alkyl chains, and three silicon atoms are distributed terminally at each alkyl chain, and each silicon atom is connected with three methoxy- groups (–OCH_3_). The ST molecules can subsequently connect with the MFI frameworks by the many covalent bonds of Si–O–Si. The ST will be a stable phase of the precursors during crystallization [32]. And then the alkyl chains of ST prevent the further development of crystals, forming primary nanosized crystals with intracrystalline mesopores.

The main work of this paper is the successful synthesis of hierarchical ZSM-5 zeolites with nanosized primary crystals and narrow intracrystalline mesopore size distribution by using the ST as mesoporogen, and we employ three typical catalysis reactions involved bulky molecules to assess the influence of catalysis performance with abundant mesoporosity introducing in the catalysts.

## Methods

### Fabrication and Verification of the Soft-Template (ST)

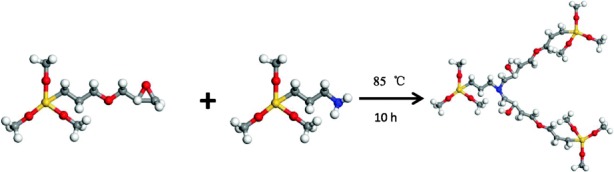



**Formula. 1.** Fabrication method of the soft-template (ST) (*white ball*, hydrogen; *gray ball*, carbon; *red ball*, oxygen; *yellow ball*, silicon; *blue ball*, nitrogen).

The mesoporogen ST is fabricated through the method of reaction by 3-aminopropyltrimethoxysilane (C_6_H_17_NO_3_Si, 179, Qufu Yi Shun Chemical Co., Ltd.) with (3-glycidoxypropyl) trimethoxysilane (C_9_H_20_O_5_Si, 236, Qufu Yi Shun Chemical Co., Ltd.) as exhibited in Formula 1. The raw materials are stirred vigorously for 10 min and then reacted in microwave chemical reactor with nitrogen protection at 85 °C for 10 h to obtain the ST product (C_24_H_57_O_13_NSi_3_, 651). The ST is simultaneously preserved in hermetic vials. The FTIR (Additional file 1: Figure S1) is used to confirm the molecular frameworks of ST.

### Synthesis of the Hierarchical ZSM-5 Zeolites

In typical synthesis method of hierarchical ZSM-5 zeolites (process 1), we added the mesoporogen ST (methanol solution, 48 wt%, *ρ* = 0.9120 g/mL; C_24_H_57_O_13_NSi_3_, molar weight 651) into the solution of 8.7 g silica sol (40 wt% SiO_2_, Guangdong Huihe Silicon Products Co., Ltd) and 20–60 mL tetrapropylammonium hydroxide (TPAOH, 25 wt%, C_12_H_29_NO, 203.37, Zhengzhou Alpha Chemical Co., Ltd), after rapid stirring, the obtained emulsion was named as A; Sodium aluminate of 0.16 g (NaAlO_2_, 82, Shanghai Kaiyun Medical Technology Co., Ltd) was added into 25 mL distilled water for 10–15 min, after rapid stirring, the obtained solution was named as B. We then added solution A to solution B, and the precursor was further stirred for 3 h. The precursor was transferred into Teflon-coated stainless-steel autoclaves and hydrothermally crystallized at 80 °C for 24 h and then 160–200 °C for 1–5 days. At last, the samples were washed and filtrated, and dried at 100 °C for 10 h and then calcined in air at 550 °C for 10 h. The typical molar composition of the precursor was 60 SiO_2_: Al_2_O_3_: 25.4–76 TPAOH: 2589–4307 H_2_O: 2–7 ST. The optimal samples in this work were produced under the crystalline conditions of 170 °C for 3 days and the optimal molar ratio of TPAOH/ST = 8, named MZ (mesoporous zeolite) in latter discussions. The amounts of the ST molecules added in MZ-1 to MZ-4 were 1.3 g, 2.2 g, 3.1 g, and 3.9 g, respectively. The detailed studies of synthesis conditions were presented in Additional file1: Figures S2–S4 and Tables S1–S3). The traditional microporous ZSM-5 zeolite, named TZ (traditional zeolite), was produced with the same method of MZ without the mesoporogen ST. The Na^+^-form samples were exchanged by 0.5 mol/L NH_4_NO_3_ at 90 °C for 1 h with a solution/sample ratio of 10 cm^3^/g, repeated for 3 times, then following the calcination at 550 °C for 6 h to obtain H^+^-form samples.

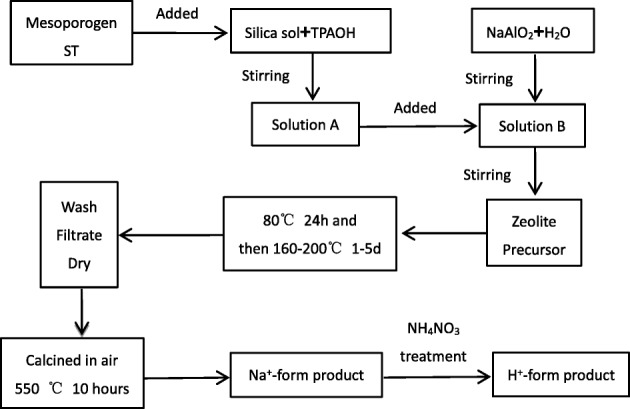



**Process 1. The preparation process of the hierarchical ZSM-5 zeolite.**

### Characterization

The Fourier transform infrared (FTIR) spectroscopy was operated on a Nicolet iS50 spectrometer. Before analysis, both samples were dehydrated in order to ensure the equal contents of adsorbed H_2_O. Powder X-ray diffraction (XRD) analysis was carried out in a Shimadzu XRD-6000 diffractometer equipped with a copper tube (*λ* = 0.15418 nm). Nitrogen sorption analysis was performed on Quantachrome Nova 2000e Surface Area & Pore Size Analyzer. Prior to analysis, all the samples were degassed at 300 °C for 10 h. The t-plot method was employed to estimate the micropore volume, and micropore surface area and external surface area. The density functional theory (DFT) method was applied to assess the mesopore size distribution. Scanning electron microscopy (SEM) images were obtained on Hitachi S4800 instrument at 10 kV. Transmission electron microscopy (TEM) images were obtained on Philips FEI Tecnai G2 F20 microscope at 200 kV. The surface acidity was operated by NH_3_ temperature-programmed desorption (NH_3_-TPD) on a Finetec Finesorb 3010 analyzer. The thermogravimetric (TG)/differential scanning calorimetry (DSC)/derivative thermogravimetric (DTG) measurements were conducted on a Netzsch Sta 449 F3 instrument. The SiO_2_/Al_2_O_3_ molar ratio was measured by the method of inductively coupled plasma (ICP) on a Varian 720 instrument. Before analysis, the samples should be pretreated by the procedures as below: first, 10 mg powder sample was placed in a plastic pipe; second, 1.6 mL acid solution (70 HCl**:** 30HF, that was consisted of 1.12 mL concentrated hydrochloric acid and 0.48 mL hydrofluoric acid) was added into the pipe with ultrasonic vibration for 15 min until all solids were completely dissolved; third, 0.6 mL concentrated nitric acid and 6 mL boric acid (5 wt%) were further added into the pipe, and then, distilled water was used to supplement the solution to total 10 mL. The hydrothermal treatment was operated at 150 °C for 10 days.

### Catalytic Reactions

The alkylation of benzene and benzyl alcohol was conducted at 80 °C with the mixture of 0.30 g sample, 68 mL benzene, and 1.0 mL benzyl alcohol. The mixture was analyzed every hour on a Varian CP3800 gas chromatography with a FID detector. Cracking of 1,3,5-tri-isopropylbenzene was conducted at 300 °C. In every injection, the amount of catalyst was 120 mg and the raw material was 0.8 μL. Before analysis, the sample was purification treated for 1 h with the N_2_ flow rate of 60 mL/min. The products were analyzed on a Varian CP3800 gas chromatography with a FID detector. The LDPE cracking reaction was conducted on a Netzsch Sta 449 F3 instrument. The LDPE was purchased from XOM (Exxon Mobil, ≤ 500 μm), density of 0.925 g/cm^3^, and fusion point of 115 °C. The H-form catalysts of 0.0023 g and the LDPE of 0.023 g were fully remixed at a crucible on the thermobalance. The air was pre-swept with the N_2_ flow rate of 60 mL/min. The cracking reaction was performed from 30 to 600 °C, and the heating rate was 10 °C/min.

## Results and Discussion

**Fig. 1:**
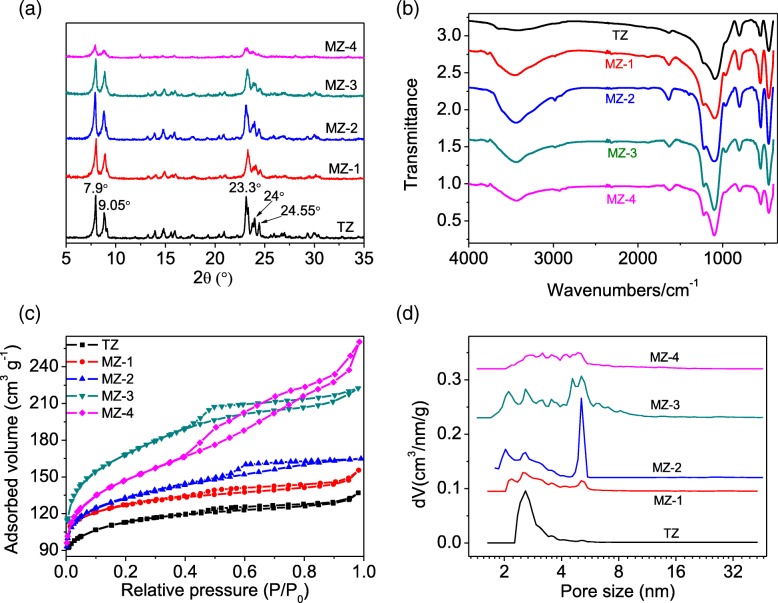
XRD (**a**), FTIR (**b**), N_2_ sorption isotherm (**c**), and mesopore size distribution (**d**) of TZ and the samples of MZ produced with different amounts of ST

The XRD spectrum of MZ samples (Fig. 1a) presents the same diffraction peaks with the sample TZ [33], further indicating that the as-synthesized MZ samples are of typical MFI structure and have high crystallinity in the presence of the mesoporogen ST. Figure 1b exhibits FTIR spectra of MZ as well as TZ for comparison. The peaks at 3490 cm^− 1^ and 1610 cm^− 1^ can be assigned to the stretching and bending vibrations of silanol groups and the adsorbed H_2_O. The peak at around 1240 cm^− 1^ is belonged to the asymmetrical stretching vibration of the external T–O [28]. The peak at about 1100 cm^− 1^ is attributed to the asymmetrical stretching vibration of the internal T–O [22]. The peaks at around 800 cm^− 1^ and 470 cm^− 1^ are belonged to the symmetrical stretching and T–O bending, respectively. The peak at 542 cm^− 1^ is attributed to vibration of the distorted DDR5 [34]. The FTIR result of the MZ samples is consistent with that of TZ, corresponding with the conclusion from the XRD analysis. The nitrogen adsorption-desorption curves and the mesopore size distributions of MZ and TZ are shown in Fig. 1c, d. The isotherms of MZ samples all exhibit an obviously hysteresis loop of typical type IV, implying the existence of mesopores [35], and in contrast, the isotherm of TZ exhibits a hysteresis loop of type I, which verifying the structure of conventional zeolites.

As shown in Table 1, the pore properties of MZ have been presented. The MZ samples were crystallined under the optimum conditions of 170 °C for 3 days and the molar ratio of TPAOH/ST = 8, and all these samples possess great data of microporosity (*S*_mic_ and *V*_mic_). According to different amounts of ST utilized, the MZ samples show different values of *S*_ext_, and as the ST increasing, the values of *S*_ext_ increase from 114 to 300 m^2^/g. The optimal amount of ST is 3.1 g of the sample of MZ-3 in this work, and the continuing increase of ST can cause the decrease of *S*_ext_ on the contrary. The optimal value of *S*_ext_ in MZ-3 reaches to 300 m^2^/g, much higher than those of almost the ever reported works in synthesizing hierarchical ZSM-5 zeolites employing different mesoporogens and methods [26, 27, 30, 36–38]. What is the most important is that the *S*_mic_ of MZ-3 can still maintain at a very high level to 316 m^2^/g, slightly lower than that of 330 m^2^/g of TZ. The hierarchy factor (HF) has been employed to estimate the hierarchical levels of zeolites [39], and the HF values of the MZ samples are 0.16–0.19, further indicating the excellent hierarchical properties. In the pore size distribution patterns, the mesopores centered at 4–8 nm can only be found on samples of MZ, strongly indicating that mesopores have been introduced in zeolite particles.

**Table 1:** Pore properties of TZ and MZ samples

Samples	*S*_BET_^a^ (m^2^/g)	*S*_mic_^b^ (m^2^/g)	*S*_ext_^c^ (m^2^/g)	*V*_mic_^d^ (cm^3^/g)	*V*_total_^e^ (cm^3^/g)	HF^f^	SiO_2_/Al_2_O_3_ ratio^g^
TZ	384	330	54	0.14	0.18	0.11	49
MZ-1	444	330	114	0.14	0.22	0.16	50
MZ-2	496	338	158	0.14	0.27	0.17	50
MZ-3	616	316	300	0.13	0.34	0.19	51
MZ-4	541	321	220	0.13	0.30	0.18	47

^a^BET surface area

^b^Micropore surface area

^c^External surface area

^d^Micropore volume

^e^Total pore volume

^f^The hierarchical factor, defined as (*V*_mic_/*V*_total_) × (*S*_ext_/*S*_BET_)

^g^The SiO_2_/Al_2_O_3_ ratio was determined by ICP method

**Fig. 2:**
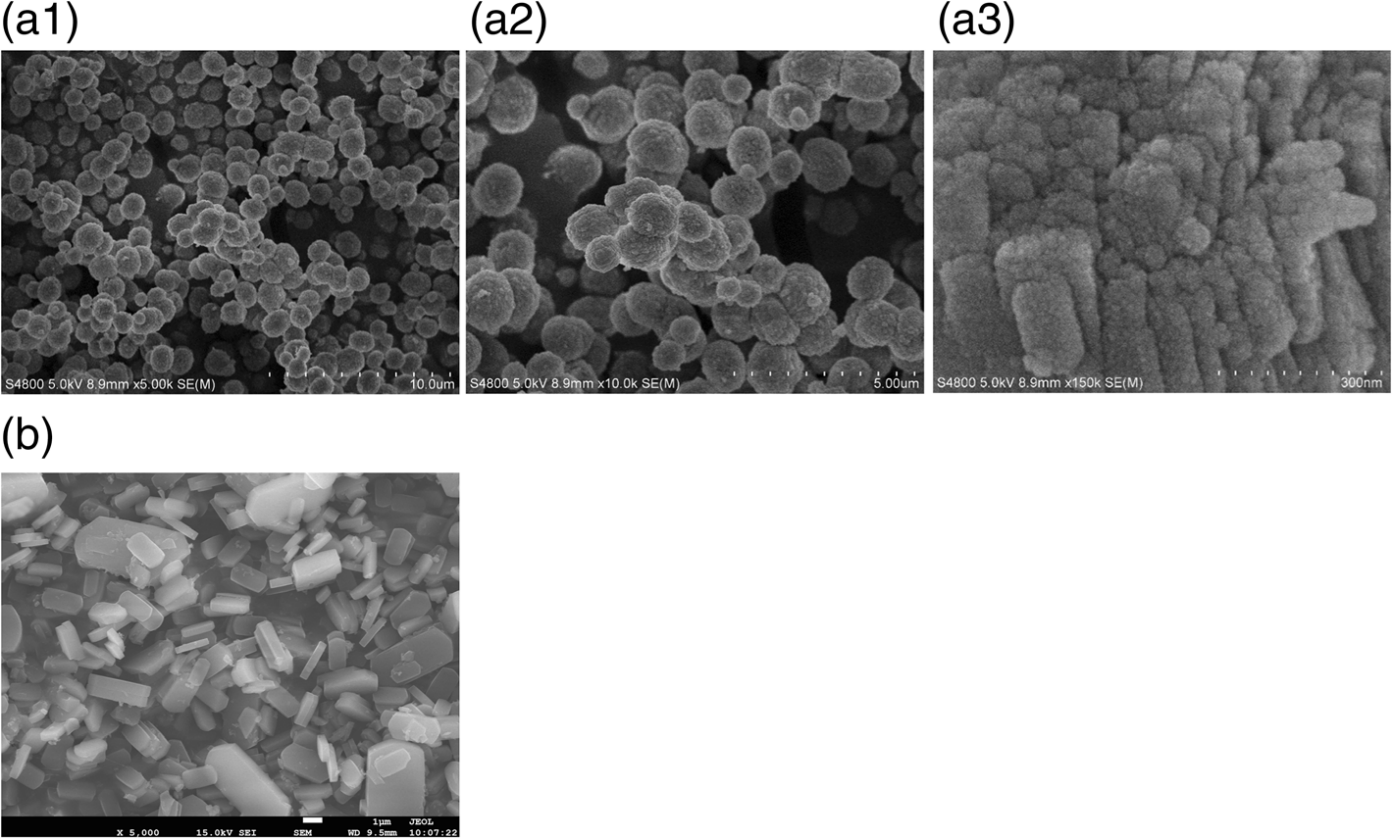
SEM images of MZ-3 (**a**) and TZ (**b**)

The morphology properties of MZ-3 have been presented in Fig. 2a1–a3. The sample of MZ-3 is made up of a large number of particles (about 1 μm) with a rough surface. And from the careful observation in Fig. 2a3, it can be found that the “coarse surface” are actually aggregations of a large number of nanocrystals with diameters of 60–150 nm. In addition, we can deduce that there are few intercrystal mesopores formed between these nanocrystals, because the mesopore size distribution is only 4–8 nm and no distributions of large pores are detected (Fig. 1d), which keep good agreement with the sorption isotherm of MZ-3 in Fig. 1c where the hysteresis loop keeping flat instead of shifting upwards at high *P*/*P*_0_ region, and the *V*_total_ of the sample MZ-3 in Table 1 is not proportionable increased in general compared with that of reported works with intercrystal mesopores [40]. All these analyses strongly prove that the mesopores built in this work are of intracrystalline property. For comparison, the morphology of sample TZ is also characterized in Fig. 2b, where typical coffin-like particles with length of 2–10 μm and width of 1–3 μm have been observed.

**Fig. 3:**
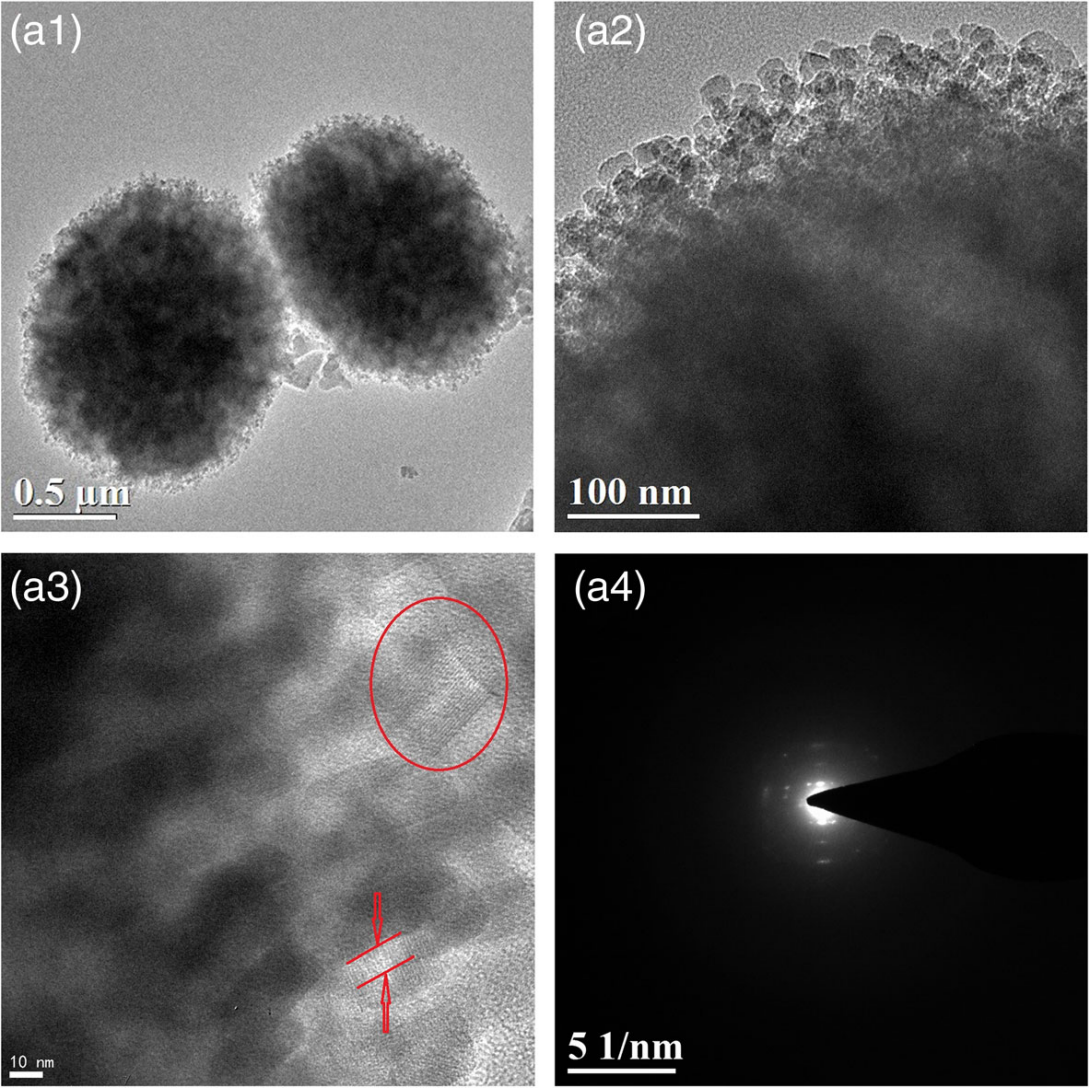
TEM images of MZ-3 (**a1**–**a3**) and selected area electron diffraction (**a4**)

The TEM images of MZ-3 are presented in Fig. 3. The image of (a1) displays the highly coarse edge, and the homogenous nanocrystals also appear in image (a2), which corresponds with the SEM observations in Fig. 2. From the HRTEM image (a3), the lattice fringes belonging to zeolite framework can be observed obviously, demonstrating the zeolites structure property of MZ-3. The SAED image (a4) has been utilized to clearly demonstrate the MFI frameworks of MZ-3. However, on account of the incapability to penetrate the whole particles, it is not obviously to exhibit the intracrystal mesoporosity and instead with dimmed and brightened spots as shown in image (a3).

**Fig. 4:**
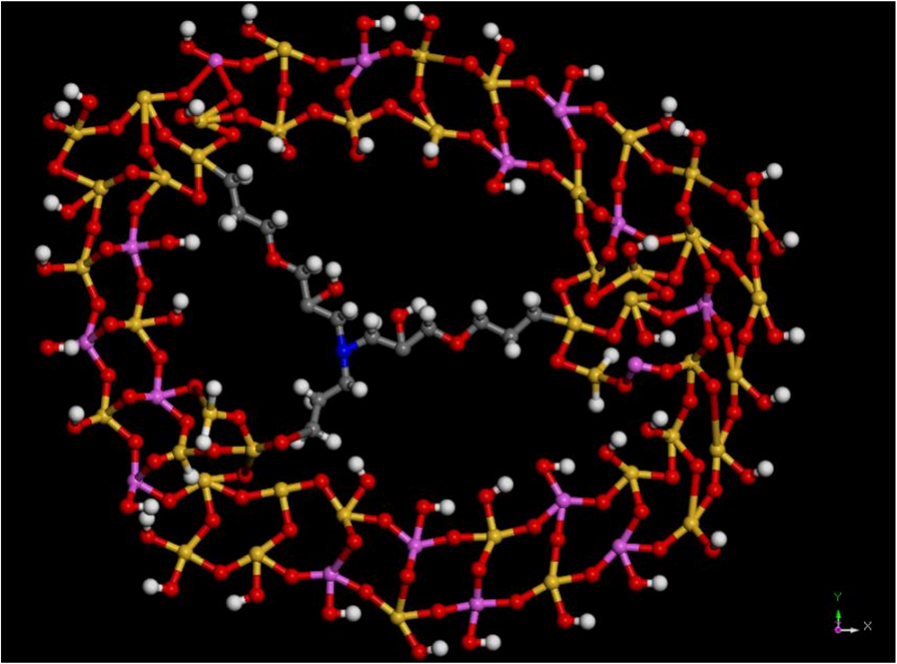
Atomic connection mode of ST with zeolite frameworks (white ball, hydrogen; gray ball, carbon; red ball, oxygen; yellow ball, silicon; magenta ball, aluminum; blue ball, nitrogen)

The atomic connection mode of ST molecule with zeolite frameworks is illustrated in Fig. 4. Due to the property of electric neutrality, the ST molecule can connect with zeolite framework with –Si–O–Si– covalent bond (condensation from –Si–OCH_3_ and –Si–OH) and has no influence on aspects of charge density and distribution or compensation. From the illustration in Fig. 4, it is easy to form a “cavity” through the branches of organic alkane chains with the method of physical space blocking, and these cavities finally form mesopores after calcination. The self-condensation also could happen between the ST molecules, which might result in different size of cavities, thus different pore size distributions of mesopores. Instead of pure physical mixture of mesoporogen with synthesis precursor to form irregular mesopores, this mesoporogen type and the connection mode could produce hierarchical zeolites with relative narrow mesopore size and homogeneous particles with great crystallinity.

**Fig. 5:**
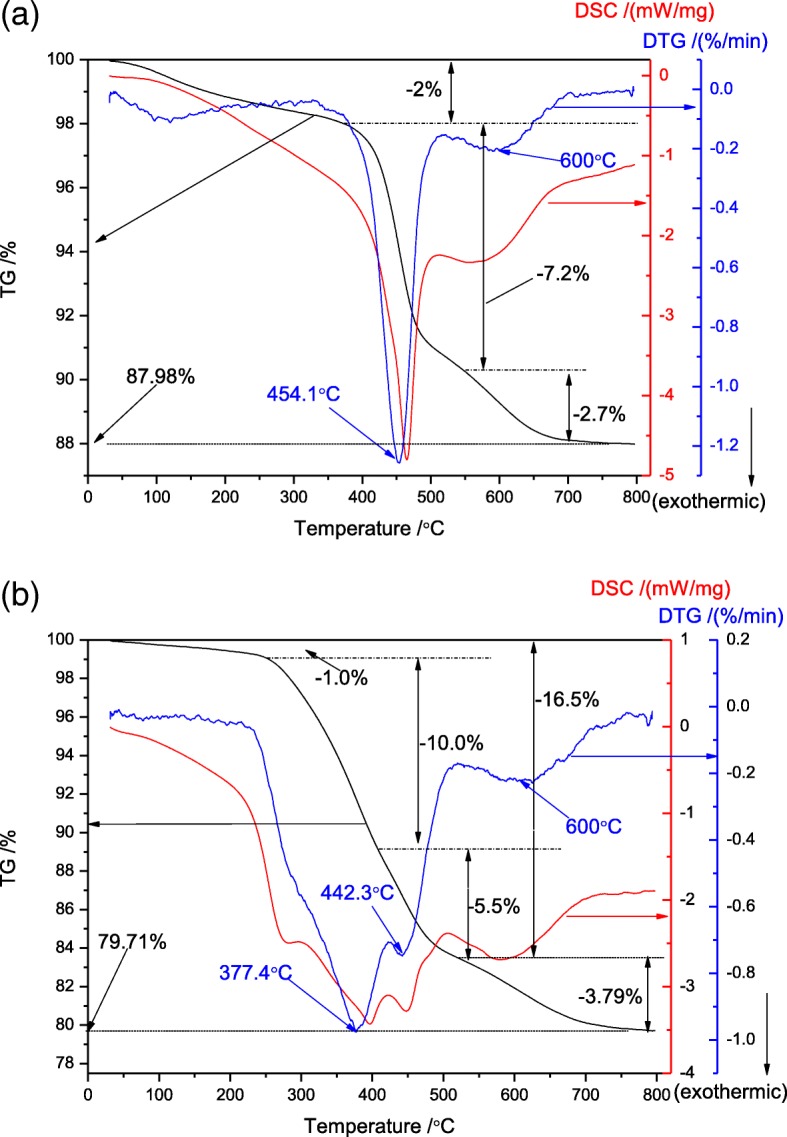
TG-DSC/DTG curves of TZ (**a**) and MZ-3 (**b**)

As shown in Fig. 5, the uncalcined samples of TZ and MZ-3 have been analyzed by thermal gravimetric. We can classify the weight loss into three regions in the curves of TZ at 50–350 °C, 350–550 °C, and 550–800 °C, attributing to remove of H_2_O of 2.0%, burning of TPA^+^ of 7.2%, and combustion of TPA^+^ occluded in occlusive cages of 2.7%, respectively [41]. The DSC and DTG curves of TZ almost show the same two peaks around 460 and 600 °C, corresponded with the decomposition of TPA^+^ [42]. From the thermal gravimetric analysis of TZ, the TG curve of MZ-3 is divided to four steps: at first, 1.0% between 50 and 255 °C, belonging to remove of H_2_O; and then, 10.0% between 255 and 405 °C, should be ascribed to the combustion of the mesoporogen ST; at last, 9.29% between 405 and 800 °C, corresponding to decomposition of TPA^+^ located in various cages. Moreover, it should be pointed that there are two peaks of the DSC curve located at 265 and 390 °C, may ascribe to the combustion of the hydrocarbon moiety and the tertiary amine moiety on ST structure, respectively. And it is reasonable that the weight loss in 350–770 °C of the samples of MZ-3 and TZ are almost the same, because of the same amounts of TPAOH utilized.

**Fig. 6:**
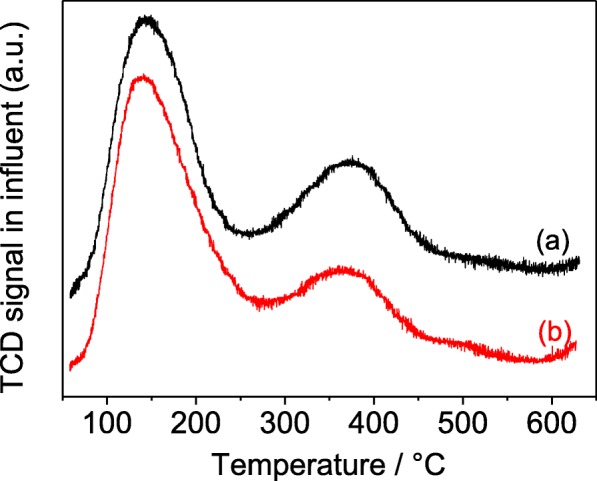
NH_3_-TPD curves of the H-form of TZ (**a**) and MZ-3 (**b**)

Figure 6 shows the NH_3_-TPD patterns of H-type TZ and MZ-3 with the same SiO_2_/Al_2_O_3_ molar ratios (SiO_2_/Al_2_O_3_ = 50). The samples all display similar curves: the peak at around 150 °C and the peak at around 375 °C, which are belonged to the weak and strong acid sites, respectively [43]. The peak located around low-temperature region can be ascribed to the interaction between hydrogen bond and silicon-oxygen bond, and the peak around high-temperature position is relevant to the framework aluminum [44, 45]. It is obviously that the amount of acid sites of sample MZ-3 is almost the same with TZ whether strong or weak acid sites, demonstrating that the acidity is correlated with the SiO_2_/Al_2_O_3_ molar ratios. These results imply that the mesoporogen ST employed in this work has been perfectly grafted to the frameworks of ZSM-5 crystal with little destruction of the surface acidity.

**Fig. 7:**
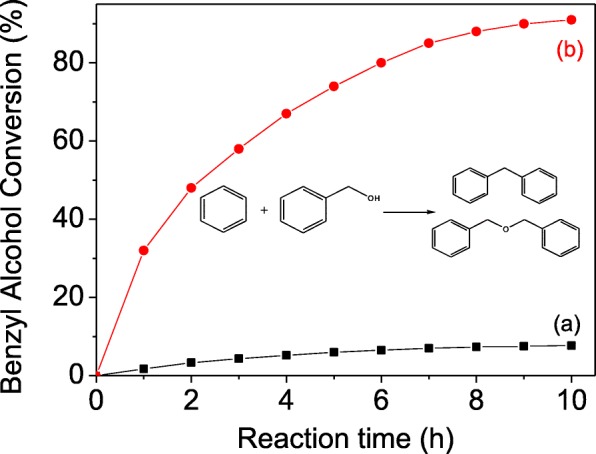
Benzyl alcohol conversions over TZ (**a**) and MZ-3 (**b**)

The alkylation between benzene and benzyl alcohol is utilized to assess the catalytic performance. The benzyl alcohol conversions over the samples of TZ and MZ-3 are recorded in Fig. 7, where traditional sample TZ exhibits very low conversion of less than 8% and the catalyst begins the deactivation after reaction for 7 h, resulting from the serious limitation of the narrow micropores and length diffusion path. On the contrary, the conversion on the sample MZ-3 can reach to 30% after reaction only for 1 h, and the catalyst still can maintain superior activity after 10 h with total conversion of more than 90%. On account of the same SiO_2_/Al_2_O_3_ molar ratio and the similar acidity, the excellent performance of MZ-3 can be resulted from the mesoporosity in crystals [46].

**Fig. 8:**
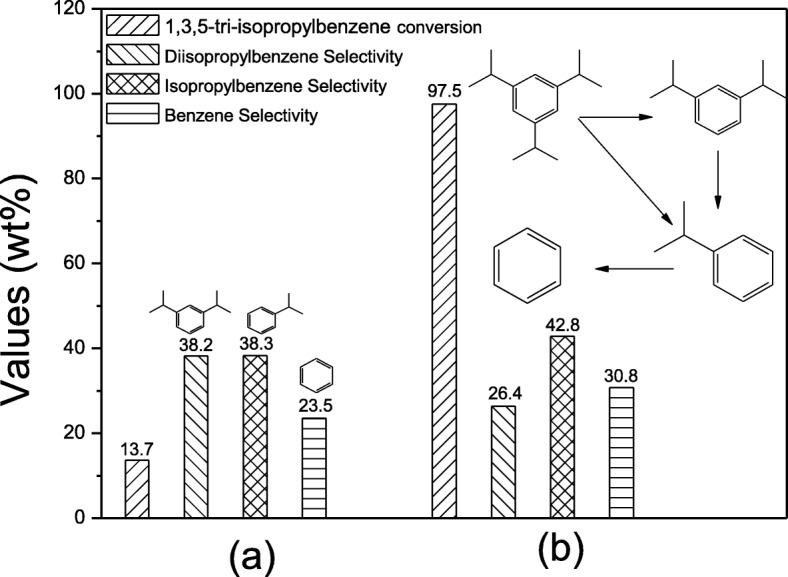
Cracking reaction of 1,3,5-tri-isopropylbenzene over TZ (**a**) and MZ-3 (**b**)

The cracking reaction of 1,3,5-tri-isopropylbenzene has been employed to estimate the catalytic performance of the hierarchical sample MZ-3. As shown in Fig. 8, this cracking reaction over zeolite catalysts is recognized with three procedures [47]. At first, 1,3,5-tri-isopropylbenzene was cracked to diisopropylbenzene (DIPB) and isopropylbenzene (IPB), and then, DIPB was cracked to IPB; at last, IPB was cracked to benzene finally.

It is significantly that the conversion of this bulky reactant on MZ-3 comes up to 97.5%, and the selectivity of product benzene is 30.8%, and the hierarchical ZSM-5 catalysts with lower SiO_2_/Al_2_O_3_ molar ratio however exhibit the selectivity of benzene of only 8.1% [48]. This result states the necessity of abundant mesoporosity, which can efficiently relieve the diffusional limitations and speed up the molecules transportation inside the crystals.

Benzene is produced in the third step, so the high selectivity of benzene on sample MZ-3 indicates the deeply cracking of intermediate products (DIPB and IPB), which also means the long resident time of intermediate products in catalyst [26], maybe due to the appropriate mesopore size (4–8 nm) in crystals. As we know, although larger mesopores benefit the diffusion of the reactants, the products have no transport limitations as well, contributing to the selectivity of primary cracking products.

On the other hand, because of the microporous structure, the reactants have no ability to enter into MFI cages; thus, it is impossible to take advantage of the acidity the inner micropores [48, 49], the conversion is only 13.7% on the sample TZ. However, although few mesopores in crystals, the sample TZ exhibits high selectivity of benzene (23.5%) and IPB (38.3%). Therefore, it is easy to deduce that the selectivity of products and the conversion of reactants can all be influenced by either the accessible acidity or the mesopore size distribution in this cracking reaction.

**Fig. 9:**
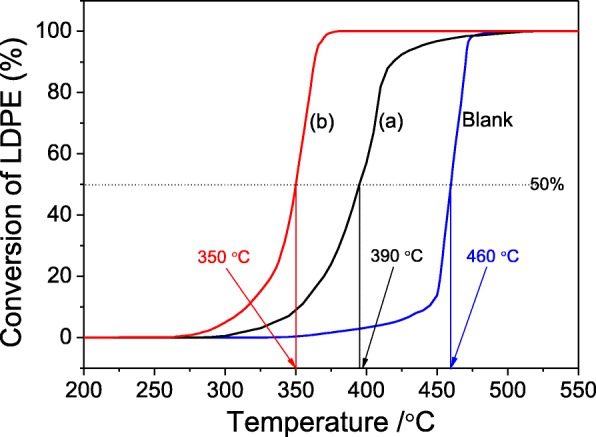
TG curves of LDPE thermal cracking (blank) and catalytic over TZ (**a**) and MZ-3 (**b**)

Diffusion limitation is a severe problem during the heterogeneous catalysis cracking reaction, and it can be improved by inducing the formation of mesopores in zeolite crystals. The LDPE thermal cracking reaction is employed to assess the diffusion ability of bulky molecules in hierarchical sample MZ-3 as shown in Fig. 9. Because the diameter of branched polyethylene chain (0.494 nm) [50] is slightly smaller than the MFI micropore size (0.5 × 0.55 nm), the LDPE cracking can be utilized to evaluate the catalytic performance on the microporous sample TZ as well, and the blank test without any catalysts is also conducted for comparison. The *T*_50_ (temperature for 50% conversion of LDPE) of blank test, TZ and MZ-3 are 460, 390, and 350 °C, respectively, implying the importance of the great diffusion superiority of polymer molecules and the extraordinary *S*_ext_ (300 m^2^/g) with abundant surface acid sites in the nanocrystals of MZ-3 [33]. The conversion of LDPE on sample MZ-3 reaches to 100% at 375 °C, and the 100% conversion on sample TZ is nearby 500 °C, which further demonstrates that the sample MZ-3 has an outstanding resistant ability of carbon deposition. In contrast, the slightly flat curve on sample TZ from 400 to 500 °C illustrates that the catalyst maybe undergo a process of decline in catalytic activity, due to the carbon deposition in the micropores.

**Fig. 10:**
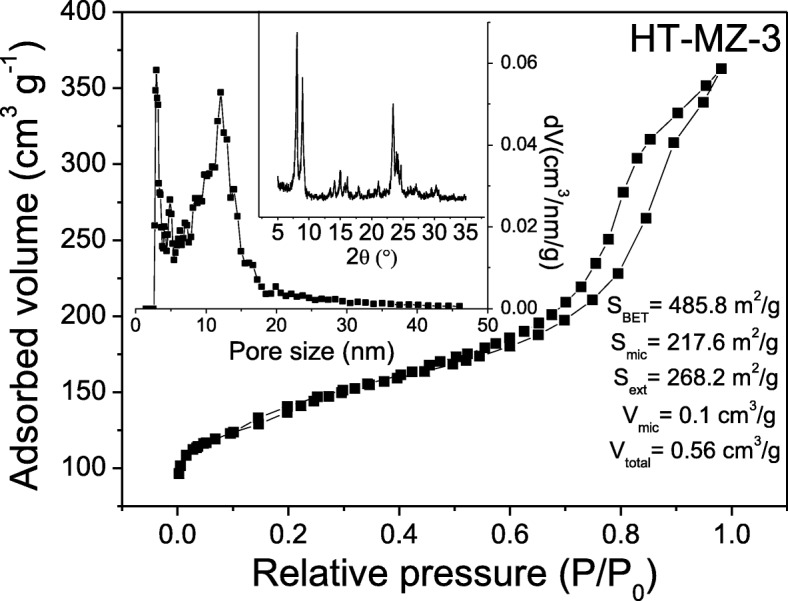
N_2_ sorption isotherm, mesopore size distribution, and XRD pattern of HT-MZ-3 (hydrothermal treated MZ-3)

**Fig. 11:**
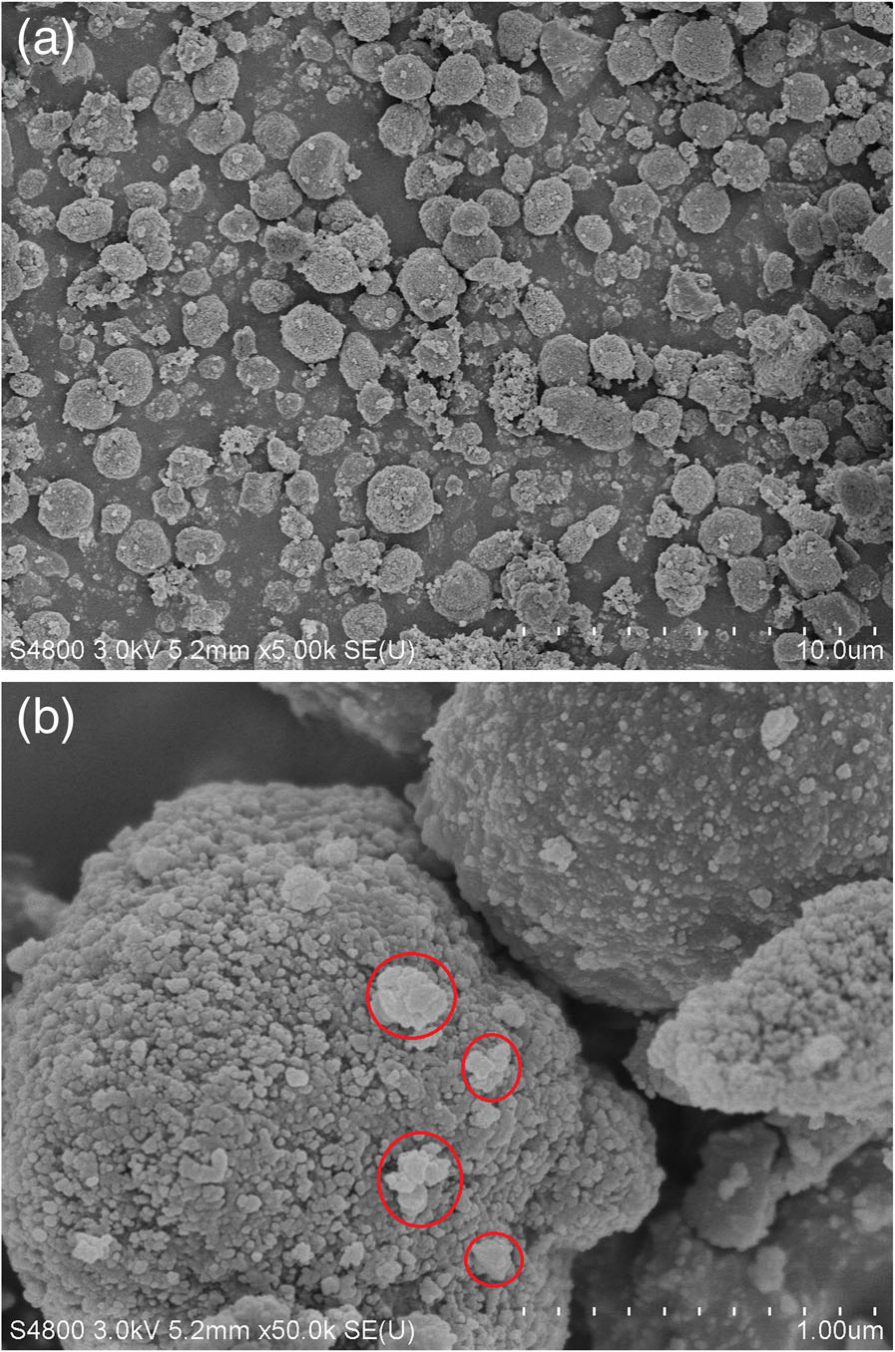
**a**, **b** SEM images of HT-MZ-3

The hydrothermal treatment (150 °C for 10 days) of the sample MZ-3 results in collapse of micropores to some extent, and the value of *S*_mic_ of HT-MZ-3 reduces to 217.6 m^2^/g as shown in Fig. 10, where the *S*_ext_ and *S*_BET_ decrease to 268.2 and 485.8 m^2^/g, respectively. This result should be ascribed to the long time of treatment and the large external surface area of MZ-3. It is known that conventional zeolites have excellent hydrothermal stability due to their pure microporous structure, and it is demonstrated that hierarchical ZSM-5 zeolites with smaller external surface area have stronger resistance to hydrothermal treatment [46]. From the analysis of the mesopore size distribution (10–20 nm) and the value of *V*_total_ (0.56 cm^3^/g), we can draw the conclusion that the intercrystalline mesopores have been created between these nanocrystals. The XRD pattern in Fig. 10 exhibits the decrease of crystallinity of HT-MZ-3, and as the collapse of micropores, the amorphous materials can be found in the SEM images in Fig. 11b. Nevertheless, the HT-MZ-3 still preserve the typical MFI crystal structure and maintain the basic morphology of the particles in MZ-3 after this severe test, further demonstrating the stability of the hierarchical structure induced by the novel soft-template (ST).

The recycle ability was also very important property of heterogeneous catalyst. We also have characterized this property by employing of the sample of MZ-3 and the reaction of cracking of 1,3,5-tri-isopropylbenzene. Twenty sets of cracking reaction were conducted consecutively without changing of the catalyst of MZ-3. After a continuous measurement in fixed bed reactor, the conversion of raw material (1,3,5-tri-isopropylbenzene) dropped to 31.8% and the selectivity of benzene was only 21.6% (Additional file 1: Figure S5) which was similar to the results obtained over conventional ZSM-5 of 23.5% in Fig. 8. The catalyst of MZ-3-used was characterized with TG method, and the total amount of coke deposition was 18.57 wt% as shown in Additional file 1: Figure S5. After TG analysis, the MZ-3-used catalyst was further characterized with SEM method as shown in Additional file 1: Figure S6, and there was almost not much change of the morphology of particles compared to that in Fig. 2. And then N_2_ sorption analysis was also utilized to characterize the pore properties of MZ-3-used directly after calcination of coke deposition in Additional file 1: Figure S7. Almost the same pore parameters have been listed in Additional file 1: Table S4, and the slightly larger values of *S*_BET_ and *S*_mic_ of MZ-3-used than that of MZ-3 could be attributed to the higher temperature in TG treatment than the calcination temperature. From the comprehensive analysis above, it is demonstrated distinctly that the hierarchical ZSM-5 zeolites obtained in this paper in the presence of mesoporogen ST was of great recycle stability.

Moreover, in order to demonstrate the outstanding mesoporosity and the excellent catalytic performance of the hierarchical ZSM-5 zeolites obtained in this paper finally, we have studied many relevant papers for comparison [51–57]. And the detailed research and comparison could be found in Additional file 1: Tables S5–S10 and Figures S8–S10), where the results have greatly evidenced the superior mesoporosity and its advantages in helping to improve the catalytic performance.

## Conclusions

The hierarchical ZSM-5 zeolites with remarkable mesoporosity have been produced in the presence of the soft-template (ST) which is simply fabricated in this work. Because of mild physicochemical and electric neutrality properties of ST molecules, these hierarchical samples possess high hierarchy factor and excellent microporosity. The intracrystalline mesopore size distribution at 4–8 nm and the abundant surface acidity greatly promote the catalytic performance in heterogeneous catalysis reaction of alkylation between benzene and benzyl alcohol, cracking reaction of 1,3,5-tri-isopropylbenzene, and thermal cracking reaction of LDPE. The as-obtained samples are proved to be efficient as catalysts in these bulky molecules involved reactions whether the conversion of reactants or the selectivity of products. And it is worth to mention that various types of soft templates can be fabricated with this route, and various types of hierarchical zeolites can also be synthesized in the presence of these mesoporogens, which can greatly expand the industrial applications in the near future.

## Additional File

Additional file 1: Electronic Supplementary Information. (DOC 20000 kb)

## Abbreviations

DFT: Density functional theory
DSC: Differential scanning calorimetry
DTG: Derivative thermogravimetric
FTIR: Fourier transform infrared
HF: Hierarchical factor
HT-MZ: Hydrothermal treated mesoporous zeolite
ICP: Inductively coupled plasma
MZ: Mesoporous zeolite
MZ-3-used: The MZ-3 catalyst used after 20 sets of cracking reaction of 1,3,5-tri-isopropylbenzene continuously
SEM: Scanning electron microscopy
ST: Soft-template
TEM: Transmission electron microscopy
TG: Thermogravimetric
TPD: Temperature-programmed desorption
TZ: Traditional zeolite
XRD: X-ray diffraction
