# Supplementary material for: Synthesis and Characterization of Hierarchical ZSM-5 Zeolites with Outstanding Mesoporosity and Excellent Catalytic Properties
Source: Nanoscale Res Lett. 2018 Nov 15;13:364. doi: 10.1186/s11671-018-2779-8 (PMC6237675; doi:10.1186/s11671-018-2779-8)
Supplement: Supplementary file 1 — Electronic Supplementary Information. (DOC 20000 kb) [file 11671_2018_2779_MOESM1_ESM.doc]

**<Supplementary Information>**

**Synthesis and Characterization of Hierarchical ZSM-5 Zeolites with Outstanding Mesoporosity and Excellent Catalytic Properties**

Guoqiang Song ^1, 2, 3^, Wenting Chen ^2^, Peipei Dang ^2^, Shengyuan Yang ^2^, Yuan Zhang ^2^, Yuanyi Wang ^2^, Ruidi Xiao ^2^, Rong Ma ^2^, and Fuxiang Li ^4, *^

^1^ 2011 Special Functional Materials Collaborative Innovation Center of Guizhou Province, Guizhou Institute of Technology, 1st Caiguan Road, Yunyan District, Guiyang City 550003, Guizhou Province.

^2^ Engineering technology research center of fluorine silicon material, School of chemical engineering, Guizhou Institute of Technology.

^3^ Key Laboratory of Light Metal Materials Processing Technology of Guizhou Province, Guizhou Institute of Technology.

^4^ College of chemistry and chemical engineering, Taiyuan University of Technology, Taiyuan 030024, Shanxi Province.

* Corresponding authors: [l63f64x@163.com](mailto:L63f64x@163.com); Tel. : (+86) 0351-6111178

Figure. S1 The FTIR spectrum of ST.

The FTIR spectrum of ST shows the disappearances of the absorption bands at 900 cm^-1^ and 3500-3300 cm^-1^, which can be assigned to the [epoxy group](http://www.iciba.com/epoxy_group)s in the (3-glycidoxypropyl) trimethoxysilane reagent and the amino groups in the 3-aminopropyltrimethoxysilane, respectively. This FTIR result confirms that the epoxide ring open to form hydrolytically stable C-N bonds with the amino groups showed in Formula. 1, and consequently obtain the organosiloxane product.

Figure. S2 The N_2_ sorption isotherms (A), and the corresponding pore size distributions (B) and the XRD patterns (C) of the samples synthesized with different TPAOH adding amounts

Table. S1 The pore parameters of the samples synthesized with different TPAOH adding amounts

| Sample | Total BET surface area  (m^2^ g^-1^) | Micropore surface area  (m^2^ g^-1^) | Mesopore surface area  (m^2^ g^-1^) | Micropore volume  (cm^3^ g^-1^) | Total pore volume  (cm^3^ g^-1^) | TPAOH adding amounts (mL) | ST adding amounts (mL) |
| --- | --- | --- | --- | --- | --- | --- | --- |
| a | 325 | 214 | 111 | 0.09 | 0.21 | 20 | 5 |
| b | 432 | 298 | 134 | 0.13 | 0.27 | 30 |
| c | 496 | 338 | 158 | 0.14 | 0.27 | 40 |
| d | 492 | 339 | 153 | 0.14 | 0.29 | 50 |
| e | 478 | 351 | 127 | 0.15 | 0.28 | 60 |

Figure. S3 The N_2_ sorption isotherms (A), and the corresponding pore size distributions (B) and the XRD patterns (C) of the samples synthesized with different crystallization temperature.

Table. S2 The pore parameters of the samples synthesized with different crystallization temperature

| Sample | Total BET surface area  (m^2^ g^-1^) | Micropore surface area  (m^2^ g^-1^) | Mesopore surface area  (m^2^ g^-1^) | Micropore volume  (cm^3^ g^-1^) | Total pore volume  (cm^3^ g^-1^) | Crystallization  temperature (℃) | ST adding amounts (mL) |
| --- | --- | --- | --- | --- | --- | --- | --- |
| a | 448 | 289 | 159 | 0.12 | 0.28 | 140 | 7 |
| b | 502 | 310 | 192 | 0.13 | 0.30 | 160 |
| c | 616 | 316 | 300 | 0.13 | 0.34 | 170 |
| d | 485 | 338 | 147 | 0.13 | 0.29 | 180 |

Figure. S4 The N_2_ sorption isotherms (A), and the corresponding pore size distributions (B) and the XRD patterns (C) of the samples synthesized with different crystallization time.

Table. S3 The pore parameters of the samples synthesized with different crystallization time

| Sample | Total BET surface area  (m2 g-1) | Micropore surface area  (m2 g-1) | Mesopore surface area  (m2 g-1) | Micropore volume  (cm3 g-1) | Total pore volume  (cm3 g-1) | Crystallization  time  (d) | ST adding amounts (mL) |
| --- | --- | --- | --- | --- | --- | --- | --- |
| a | 438 | 295 | 143 | 0.12 | 0.26 | 1 | 5 |
| b | 490 | 349 | 141 | 0.14 | 0.27 | 2 |
| c | 496 | 338 | 158 | 0.14 | 0.27 | 3 |
| d | 496 | 342 | 154 | 0.14 | 0.26 | 4 |

From the analysis above, the optimum crystallization conditions are 170 ℃ for 3 days with molar ratio of TPAOH/ST = 8.

Figure. S5. The reaction of cracking of 1,3,5-tri-isopropylbenzene and TG curve of the catalyst of MZ-3-used after 20 sets of tests consecutively.

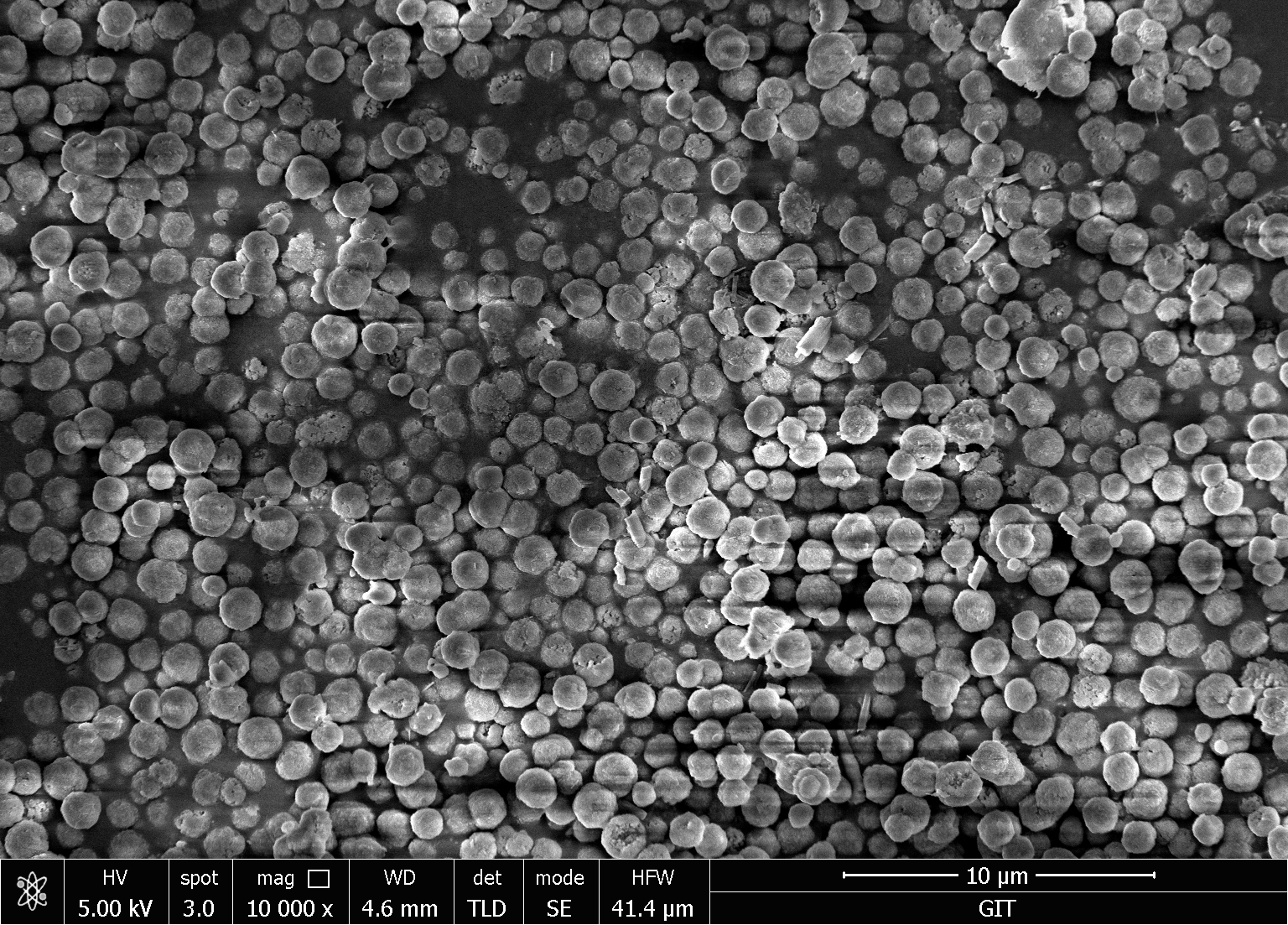

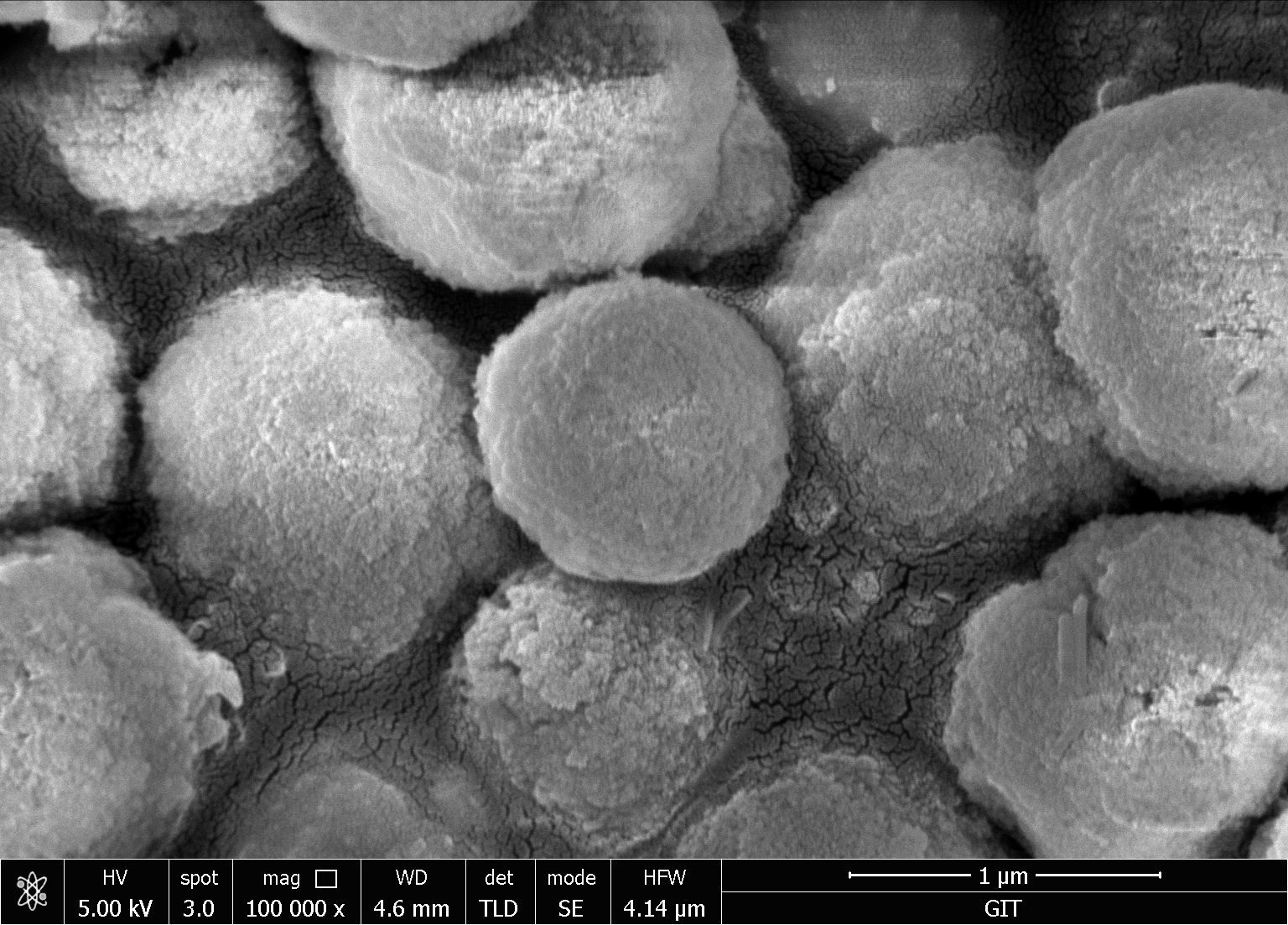

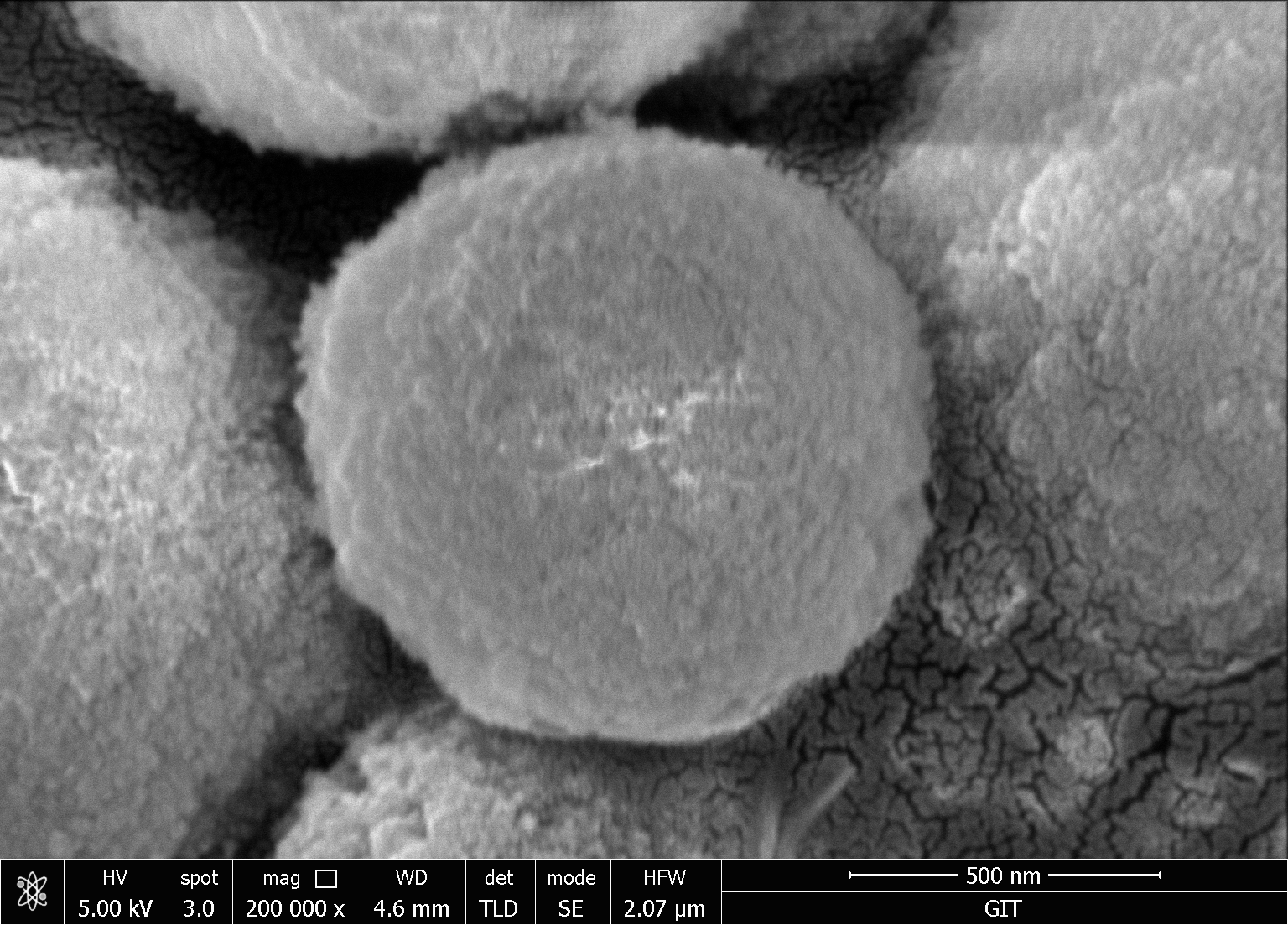

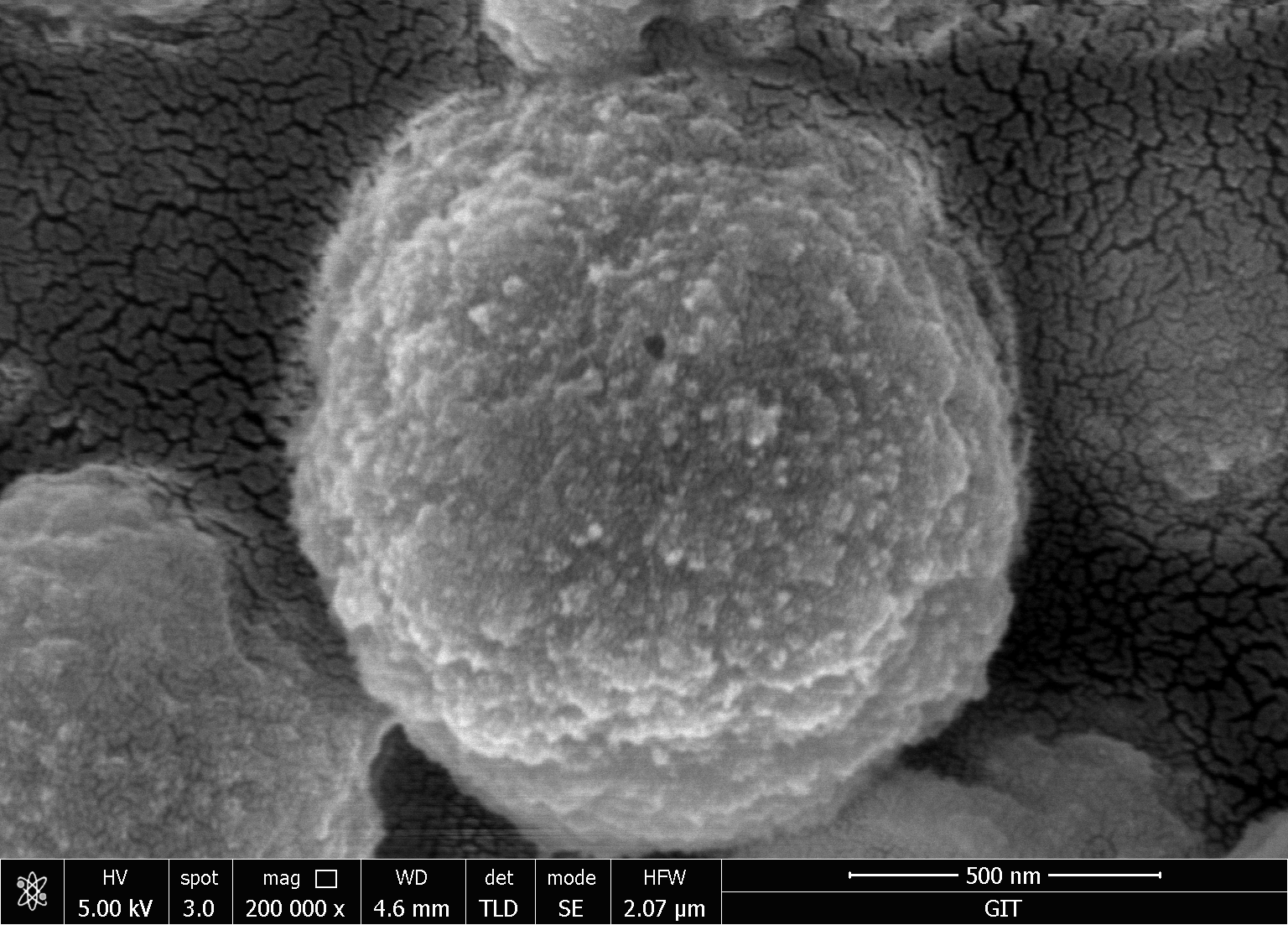


Figure. S6. The SEM image of the catalyst of MZ-3-used.

Figure. S7. The N_2_ sorption isotherms and corresponding pore size distributions of the catalysts of the MZ-3-used and MZ-3.

Table. S4 Pore properties of the MZ-3-used and MZ-3.

| Samples | S_BET_  (m^2^/g) | S_mic_  (m^2^/g) | S_ext_  (m^2^/g) | V_mic_  (cm^3^/g) | V_total_  (cm^3^/g) |
| --- | --- | --- | --- | --- | --- |
| MZ-3-used | 621 | 325 | 296 | 0.13 | 0.34 |
| MZ-3 | 616 | 316 | 300 | 0.13 | 0.34 |

Hierarchically nanoporous ZSM-5 with micropores and mesopores was successfully one-pot synthesized through a dual-template using tetrapropylammonium hydroxide and poly(ethylene oxide) as microporous and mesoporous templates, respectively in [51]. The S_BET_ of hierarchical catalyst was only 417 m^2^/g as shown in Table. S5. And the reaction of cracking of 1,3,5-tri-isopropylbenzene was utilized to characterize the catalytic activity of catalyst. The conversion of 1,3,5-tri-isopropylbenzene was just 46.5% and the selectivity of benzene was only 7.6% as shown in Table. S6.

And many other hierarchical ZSM-5 catalysts had been synthesized in presence of different mesoporogens [52-55], and the pore parameters of these samples were exhibited in Table. S7, where lower S_BET_ were presented in comparison with that of in our manuscript.

A nanosized-ZSM-5/SBA-15 analog composites (ZSC) ha been successfully synthesized in a two-step process from zeolite seed solutions containing intact ZSM-5 nanocrystals (nano-ZSM-5) by high-temperature synthesis in mild acidic media [56]. In this method, the pore properties and catalytic conversions of 1,3,5-tri-isopropylbenzene showed in Table. S8 and S9 still indicated a slightly poor performance compared to our manuscript.

Hierarchically structured zeolites (HSZs) have been successfully synthesized via a newly developed steam-assisted crystallization process in [57], where the as-obtained samples exhibited markedly enhanced catalytic performances resulting from their enlarged external surface area and shortened diffusion length in the microporous system. And from the Table. S10, it could be found that the largest S_ext_ was 428 m^2^/g of the sample HSZ30-0.1, however, the sample of HSZ30-0.1 was amorphous material indeed as the XRD pattern displayed in Fig. S8. And the highest conversion of 1,3,5-tri-isopropylbenzene was characterized over the sample of HSZ-80-0.06 as shown in Fig. S9, which was also a excellent performance similar to the sample of MZ-3 in Fig. 8, however, the pore properties of HSZ-80-0.06 in Table. S10 was not good enough and only 173 m^2^/g of S_ext_ could be detected over HSZ-80-0.06.

Table. S5 Textural parameters over hierarchically ZSM-5 and conventional ZSM-5 [51].


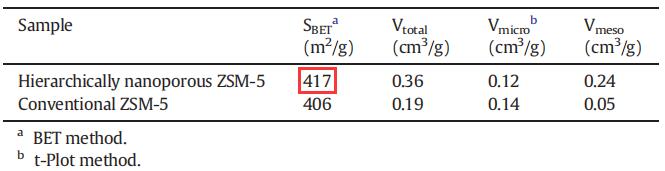


Table. S6 Catalytic activities in cracking of 1,3,5-tri-isopropylbenzene over hierarchically nanoporous ZSM-5 and conventional ZSM-5 [51].


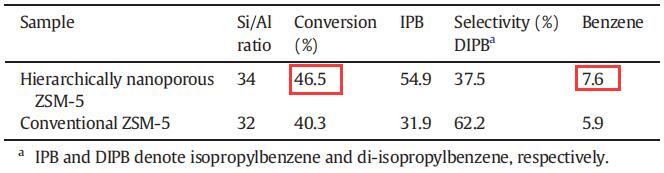


Table. S7 The porosity of mesoporous ZSM-5 synthesized with different templates. [52-55].


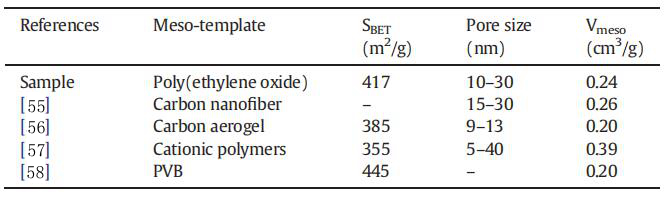


Table. S8 Structural and textural properties of ZSC composites, Al-SBA-15, and H-ZSM-5 [56].


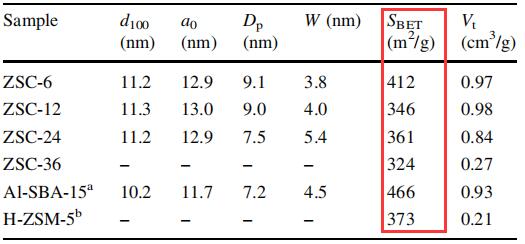


Table. S9 1,3,5-tri-isopropylbenzene conversions over various catalysts [56].

| Sample | TIPB conversion (%) |
| --- | --- |
| ZSC-6 | 78.3 |
| ZSC-12 | 80.9 |
| ZSC-24 | 82.5 |
| ZSC-36 | 40.6 |
| Al-SBA-15 | 85.2 |
| H-ZSM-5 | 61.9 |

Table. S10 Textural Parameters, Composition, and RC of All Synthesized Materials [57].


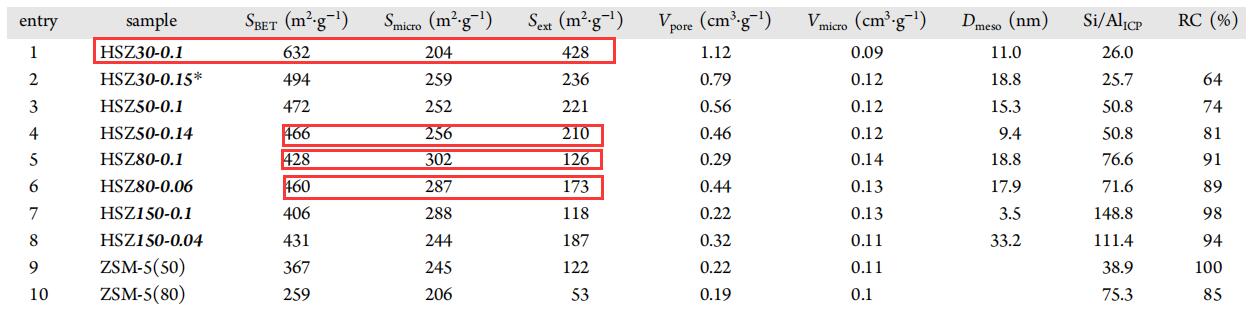


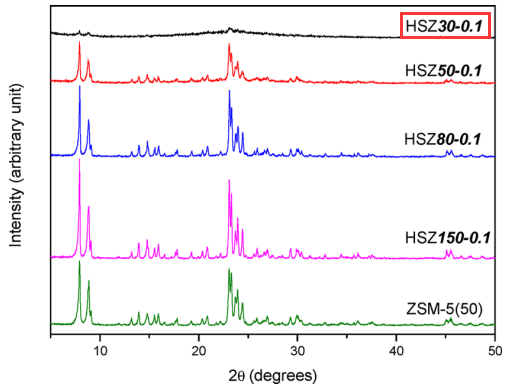


Figure. S8. XRD patterns of HSZ30-0.1, HSZ50-0.1, HSZ80-0.1, HSZ150-0.1, and ZSM-5(50) [57].


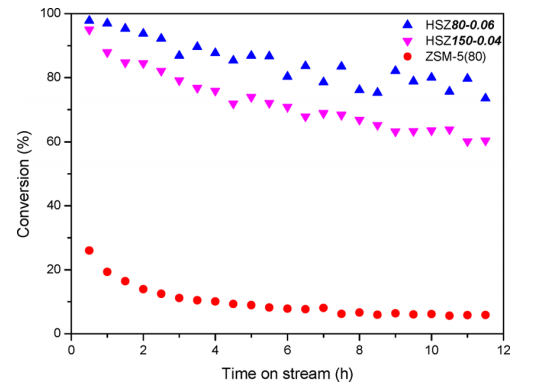


Figure. S9. Conversions of TIPB over HSZ80-0.06, HSZ150-0.04, and ZSM-5(80) during the cracking reactions [57].


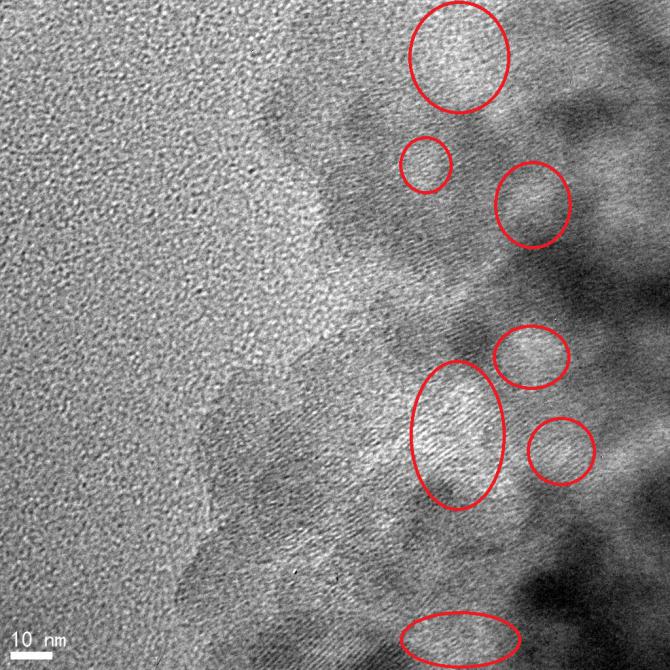


Figure. S10. The TEM image of MZ-3, and the red circles noted were lattice fringes of micropores.
